# Virulence Attenuation of *Candida albicans* Genetic Variants Isolated from a Patient with a Recurrent Bloodstream Infection

**DOI:** 10.1371/journal.pone.0010155

**Published:** 2010-04-13

**Authors:** Paula Sampaio, Marlene Santos, Alexandra Correia, Fábio E. Amaral, Julio Chavéz-Galarza, Sofia Costa-de-Oliveira, António G. Castro, Jorge Pedrosa, Célia Pais

**Affiliations:** 1 Centre of Molecular and Environmental Biology (CBMA), Department of Biology, University of Minho, Braga, Portugal; 2 Life and Health Sciences Research Institute (ICVS), School of Health Sciences, University of Minho, Braga, Portugal; 3 Faculdad de Ciencias Biológicas, Universidad Nacional de Trujillo, Trujillo, Perú; 4 Department of Microbiology, Porto Faculty of Medicine, University of Porto, Porto, Portugal; The Research Institute for Children at Children's Hospital New Orleans, United States of America

## Abstract

The incidence of *Candida albicans* infections and the relapse episodes after antifungal treatment have increased in recent decades. Recurrences are mainly due to the persistence of the original infecting strain that may present genetic and genomic rearrangements during interaction with the host, reflecting strain adaptation. In this study, four isolates recovered from a patient during recurrent candidemia episodes were genotyped by microsatellite length polymorphism (MLP) and by multilocus sequence typing (MLST) and found to be genetic variants of the same strain. Using experimental mouse infections, a progressive reduction in the virulence of the four isolates was observed, with the first two isolates more virulent than the third and fourth. Additionally, in the mouse model, the first isolate resisted host control more efficiently, resulting in higher kidney fungal burdens and necrosis as compared to the third isolate. The resolution of inflammation was delayed in mice challenged with the first isolate and the message for IFN-γ and TNF-α in the spleen was lower within the first few hours post-infection. Original and recurrent isolates also displayed different phenotypes regarding activity of secreted enzymes and response to stress agents. Overall, the comparative analysis indicated that the virulence decrease of these isolates was related to a lower ability to resist to the host anticandida effector mechanisms. We showed for the first time that *C. albicans* genetic variants of the same strain, sequentially isolated from an immunocompromised patient, underwent adaptations in the human host that resulted in virulence attenuation when tested in mice.

## Introduction

*Candida albicans* is a common colonizer of the human gastrointestinal, respiratory, and reproductive tracts. However, in immunocompromised patients, this species is one of the most important opportunistic fungal pathogens, being responsible for both superficial and systemic infections [1], [2]. Despite the prevalence of *Candida* in the hospital environment and the poor outcome of this infection, the pathways involved in clearance of mucocutaneous and systemic infections have not been fully defined and the majority of the clinical studies focus on epidemiology, diagnosis and therapeutic management [3].

Molecular epidemiology studies showed that *C. albicans* isolates exhibit a high level of genetic diversity. Microsatellite length polymorphism (MLP) and multilocus sequence typing (MLST) have been used to discriminate *C. albicans* strains and to detect small genetic changes or microvariations that may be indicative of adaptability processes [4]–[7]. Typing of multiple *C. albicans* isolates from the same patient obtained in longitudinal studies, or in surveillance cultures from different anatomical sites, showed a tendency towards the maintenance of the same strain during the infection process [6], [7]. This view of a monoclonal infecting population has recently been extended by the demonstration of colony-to-colony variation in *C. albicans* primary isolations in samples from patients with vaginal and oral infections [8]. Nevertheless, the referred study also showed that strain variability in primary cultures from established infections is much lower than from healthy individuals, suggesting that the infecting population results from the selective proliferation of one or more clones that were present in the mixed commensal population before the establishment of the infectious process. Observations on the genetic and phenotypic variation in *C. albicans* populations showed higher rates of chromosome-level genetic variations during passage in the mouse relatively to in vitro growth [9], and in strains isolated from the digestive tract of healthy individuals [10]. These genomic alterations may be involved in the generation of new variants within the population that contribute to the adaptation during infection.

Host defense against systemic candidiasis relies mainly on the ingestion and elimination of *C. albicans* by cells of the innate immune system, in particular macrophages, monocytes, and neutrophils [11]–[15]. Activation of leukocytes by *C. albicans*, triggers the release of pro-inflammatory cytokines (Th1 and Th17 responses), such as IFN-γ, TNF-α, IL-1β, IL-6, and IL-17 that in turn activate phagocyte effector functions to eliminate the invading yeast [12], [16]–[19]. In contrast, anti-inflammatory cytokines (Th2 response) such as IL-4 and IL-10 have immunosuppressive effects. Thus, the balance between pro- and anti-inflammatory cytokines is decisive in determining whether the host defense system is overwhelmed or able to eliminate the fungal pathogens [14], [20]–[25]. Although the status of the host immune system is the major factor balancing the transition from commensalism to pathogenicity [26], *C. albicans* expresses several virulence attributes that contribute for its successful behavior, both as a commensal colonizer and as a pathogen [27]. One of its major virulence traits is the ability to reversibly switch from unicellular budding cells to filamentous forms and the yeast uses this attribute during an infection, not only to invade tissues, but also to escape intracellular phagocyte death by inducing hyphal growth inside the phagosome, resulting in the destruction of the macrophage [28]–[30].

In the present work, we assessed the virulence of *C. albicans* isolates from a patient with recurrent candidemia treated during a period of four months with fluconazole. Typing of the isolates determined that they were variants of the same strain and it was observed that those genetic variants were progressively less virulent to mice. With this study, we show for the first time that variants of the same strain, recovered from a patient during recurrent infections, differ considerably in terms of their capacity to produce disease when tested in an immunocompetent host.

## Results

### *Candida albicans* isolates from a case of recurrent candidemia are genetic variants of the same strain

**Figure 1:**
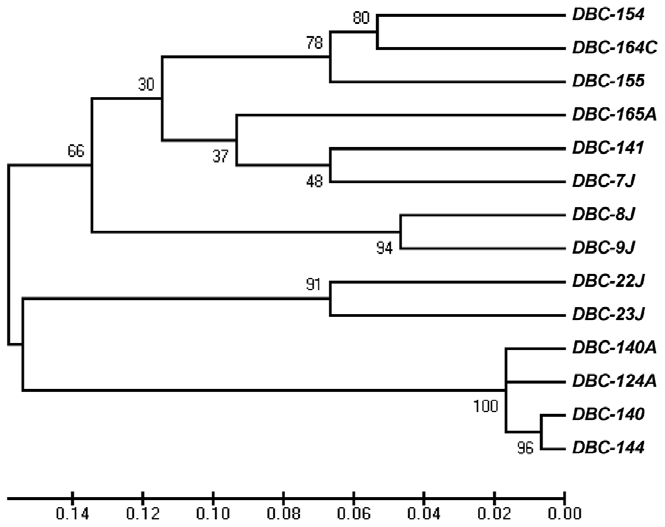
*C. albicans* strain clustering. Similarities between MLST data were analyzed in terms of p distance with MEGA version 4.0 and nodal support values, after 1000 bootstrap replications, were calculated and are depicted on the UPGMA dendrogram.

*C. albicans* isolates used in this study were recovered from cases of recurrent candidemia (Table 1). The four isolates analysed in more detail were obtained from patient 1 and collected within a period of four months. Isolate 124A was the first recovered and, despite the patient's treatment with fluconazole, three other isolates, 140, 140A, and 144, were sequentially collected. All four isolates were found to be resistant to fluconazole, presenting MIC values >64 µg/ml. These isolates showed the same multilocus genotype by MLP, except 140A, which presented a loss of heterozygosity (LOH) at CAI microsatellite (Table 1). MLST analysis also showed that the isolates were closely related, although presenting minor differences, resulting in different diploid sequence types (DSTs). To gain a better insight into the genetic proximity of these four isolates, a similarity UPGMA dendrogram based on MLST data was constructed. Strains isolated from other patients were also typed and included in the Clustal analysis to generate a more robust tree. This analysis showed that all isolates from patient 1 grouped closely, within a *p* distance value lower than 0.02, and with a nodal support value of 1 after 1000 bootstrap replications (Fig. 1), indicating that the four isolates could be considered undistinguishable, or genetic variants of the same strain.

**Table 1:** Microsatellite genotypes and diploid sequence types (DSTs) obtained by MLP and MLST analysis of the clinical isolates used in this study.

*Patient*	*Isolate*	*Isolation Data*	*Local of isolation*		*MLP Genotypes*		*MLST DST*	*Clade*
				CAI (CAA/G)_n_	CAVI (TAAA)_n_	CEF3 (TTTC)_n_(TTC)_n_		
1	DBC-124A	18-05-04	Catheter	18–34	12–12	137–139	1282	16
	DBC-140	31-06-04	Blood	18–34	12–12	137–139	1283	16
	DBC-140A	31-07-04	Bronchial secretions	34–34	12–12	137–139	1284	16
	DBC-144	26-08-04	Blood	18–34	12–12	137–139	1285	16
2	DBC-154	20-09-04	Blood	26–26	7–7	135–146	1277	4
4	DBC-141	02-08-04	Blood	12–17	7–7	126–135	1278	8
5	DBC-165A	22-10-04	Pleural fluid	21–25	9–15	131–131	1279	11
6	DBC-155	21-09-04	Blood	29–29	7–11	129–143	1280	15
7	DBC-164	22-10-04	Blood	26–28	7–7	136–145	1281	4
8	DBC-7J	-	Vaginal	30–30	19–23	126–126	1286	5
	DBC-8J	-	Vaginal	30–32	19–23	126–126	1287	1
	DBC-9J	-	Vaginal	30–32	19–23	126–126	1288	1
9	DBC-22J	-	Vaginal	23–27	18–21	135–136	1289	S*
	DBC-23J	-	Vaginal	23–27	21–21	135–136	1290	S*

The corresponding date and local of isolation, as well as clade assignment based on MSLT, are also shown.

- data unknown; DST – diploid sequence type; S* - singleton.

### Mouse virulence of the isolates decreased progressively

In view of the genetic similarity of the isolates, the question of whether they also behaved identically regarding virulence towards a healthy host was raised, and the mouse model of i.v. disseminated candidiasis was used to assess virulence.

**Figure 2:**
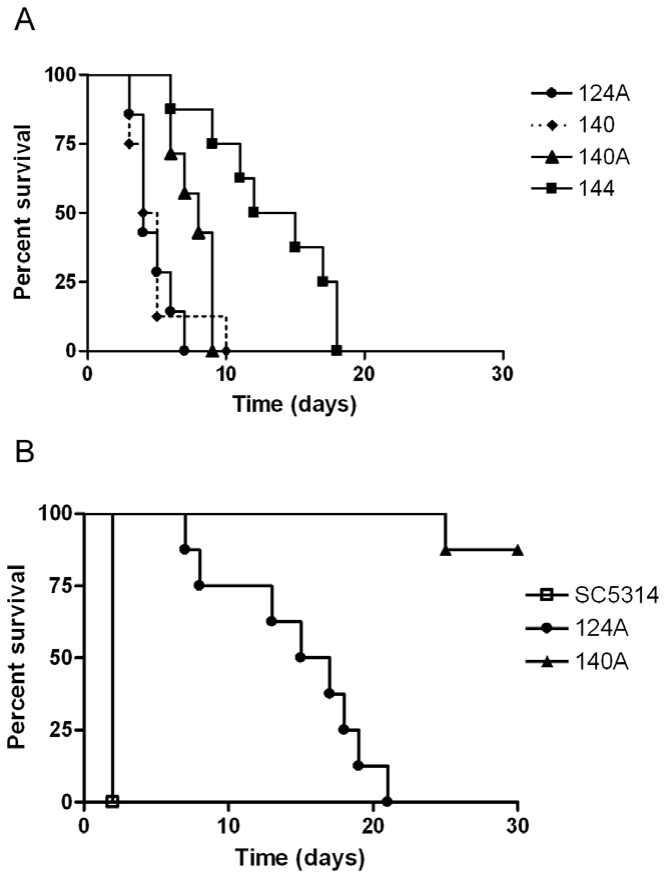
Survival of BALB/c mice following i.v. infection with *C. albicans* strain variants. Mice were infected i.v with (A) 2×10^6^ cells of isolates 124A, 140, 140A or 144 or (B) 1×10^6^ cells of SC5314, 124A or 140A and the condition of the mice were assessed daily for 30 days.

Survival analysis of mice inoculated with 2×10^6^ yeast cells showed that the first isolates (124A and 140) were the most virulent, while the last ones (140A and 144) were less virulent (Fig. 2A). Comparing mice infected with the first isolate (124A) with mice infected with the third (140A), or with the fourth (144), the overall differences in survival were highly significant (*P* = 0.0034 and *P* = 0.0002). In fact, mice infected with 124A, 140, 140A or 144 presented median survival times of 4.0, 4.5, 8.0, and 13.5 days, respectively. Differences in virulence between isolates 124A and 140A were confirmed using a lower inoculum (Fig. 2B). When testing reference strain SC5314 with the same inoculum, all mice succumbed during the first two days post-infection, in accordance to what is described in the literature [31].

These results demonstrated that genetic variants of the same strain, recovered from the same patient during recurrent infections, progressively reduced their virulence when tested in an immunocompetent host.

### Decreased survival of inoculated mice is correlated with high kidney fungal burden and necrosis

**Figure 3:**
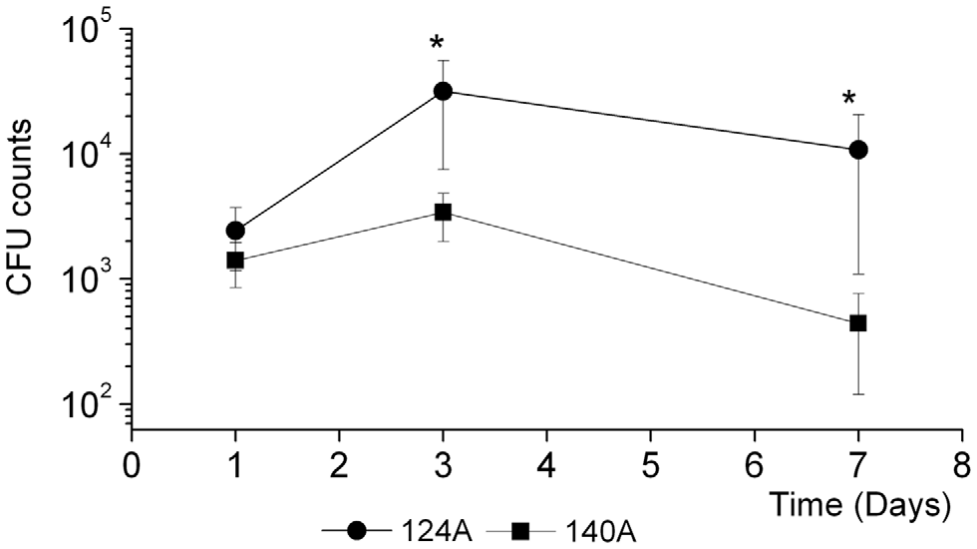
Kidney fungal burden. Groups of four mice infected i.v. with 10^6^ *C. albicans* cells were killed at 1, 3 and 7 days after challenge. Organs were homogenized in HBSS and the suspension diluted and cultured on Sabouraud dextrose agar. Results are presented as log of colony-forming units (CFUs). Statistically significant differences between results at each hour of infection as evaluated by Student's *t* test are labeled with single asterisk (*P*<0.05).

In order to understand the mechanisms underlying the differences observed in mouse survival, the most virulent isolate (124A) and the isolate with the most significant decrease in virulence (140A) were further studied. A comparative analysis of organ fungal burdens, cytokine expression and histopathology of mice inoculated with these isolates was performed up to the seventh day post-infection. Kidney fungal burden increased from days one to three, decreasing significantly on the seventh day, for mice infected with 140A (Fig 3). At day three post-infection, kidney colony counts from mice infected with 124A were around 10-fold higher when compared to mice infected with 140A, and by day seven this difference was even higher, to nearly 22 fold. On the contrary, splenic and hepatic colony counts declined progressively in all mice to nearly undetectable levels, showing no significant differences between the two isolates (results not shown). Differences regarding organ distribution are in accordance with the known predilection of *C. albicans* for kidney colonization, after mouse systemic infection [32].

**Figure 4:**
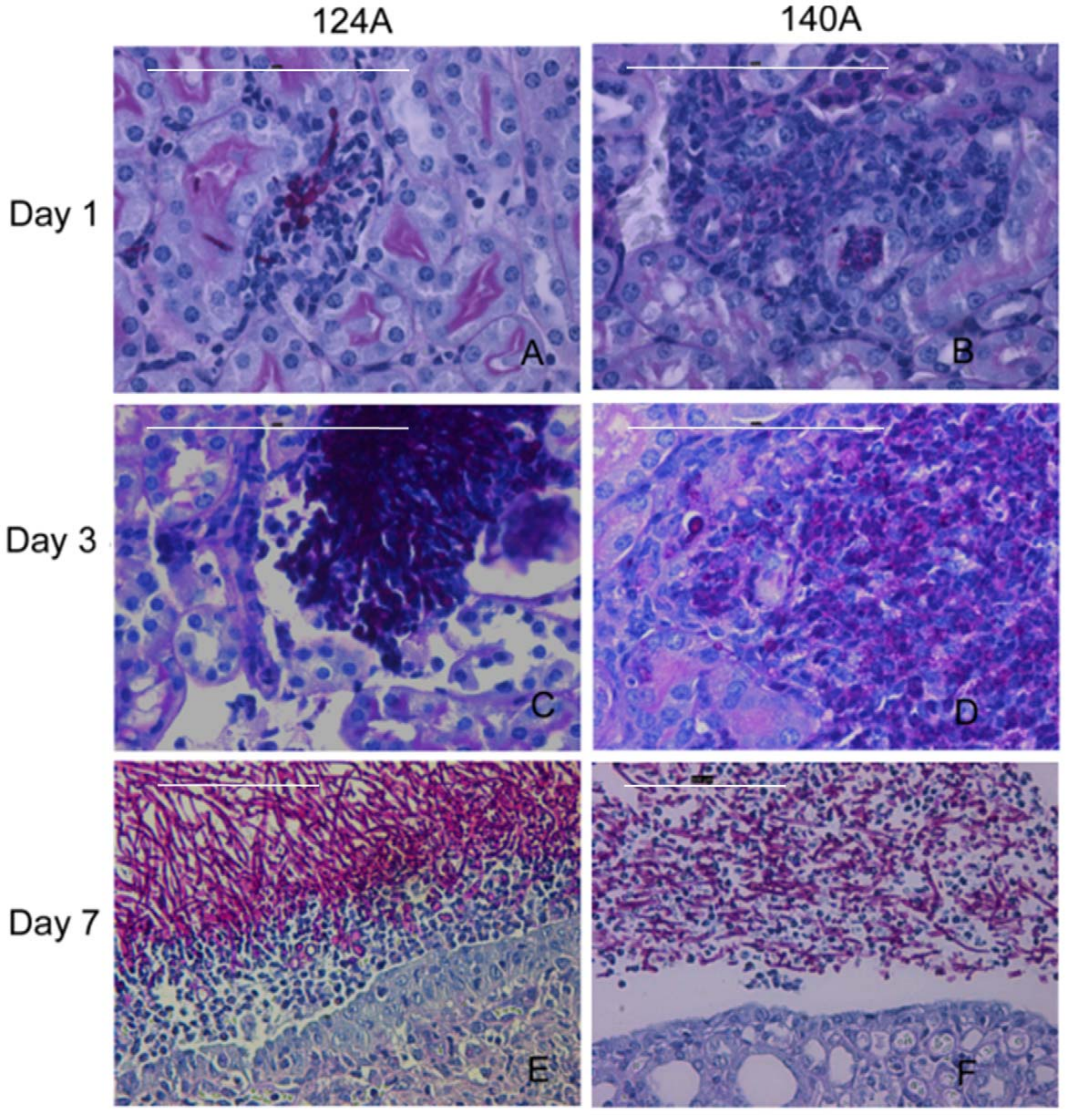
Kidney histology. Representative micrographs of H&E/PAS-stained paraffin sections of kidneys recovered from mice infected with 10^6^ yeast cells at days 1 (A and B), 3 (C and D) and 7 (E and F) days post-infection with isolates 124A and 140A.The bar −100 µM.

The higher mouse susceptibility to infection with isolate 124A was also evident in H&E and PAS stained histologic sections of the kidney (Fig. 4). At day one, and particularly at day three, kidney sections of mice infected with 124A exhibited extensive tissue necrosis and lack of an apparent cellular infiltration (Fig. 4A and 4C). Additionally, in the same period, PAS staining showed a dramatic increase in fungal cell numbers in mice infected with 124A (Fig. 4C). In contrast, kidney sections of mice infected with 140A showed degraded yeast cells, and a marked inflammatory leukocyte influx, indicating a resolving lesion (Fig. 4B and D). At day seven post-infection, in mice infected with isolate 124A, the fungal cells were predominantly in the hyphal form and were apparently intact, forming a clear barrier to the progression of inflammatory leukocytes. On the contrary, kidney histology of mice infected with 140A showed an intermixing of fungal cells with inflammatory leukocytes and degraded fungal cells, suggesting that yeast cell proliferation was controlled (Fig. 4E and F). These results are in accordance with kidney CFU counts obtained previously.

**Figure 5:**
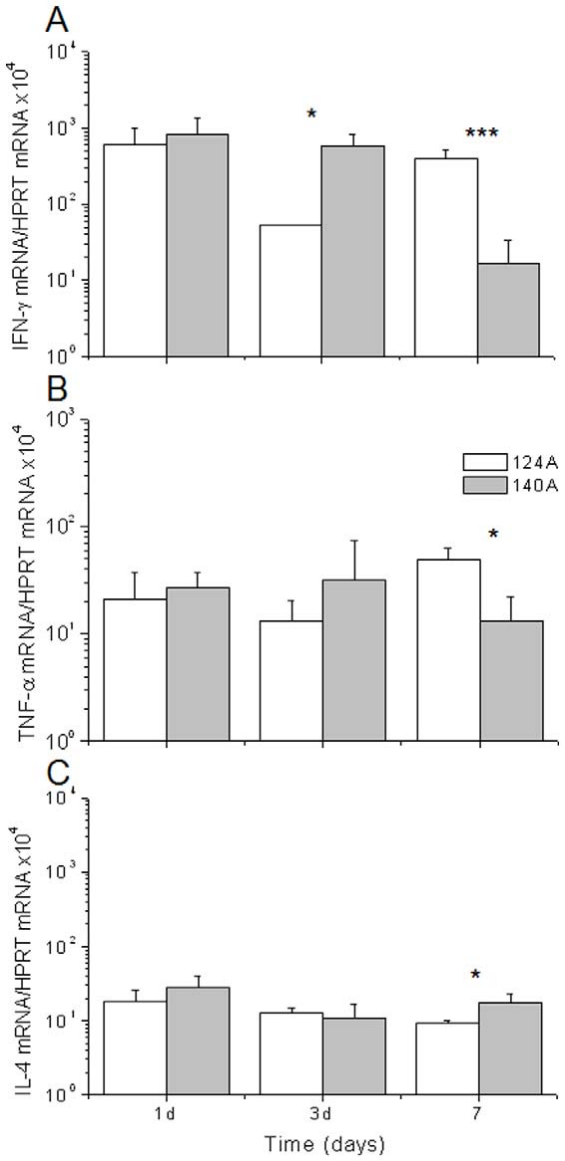
Real time PCR cytokine quantification. RNA was extracted from spleen homogenates in HBSS of mice infected with 10^6^ yeast cells of 124A (□) or 140A (▪) by using the Trizol method and mRNA levels of IFN-γ (A), TNF-α (B) and IL-4 (C) quantified and expressed as copies per HPRT gene. Statistical significance was calculated by using Student's *t* test and significant differences are labeled with a single asterisk (*P*<0.05) or triple asterisks (*P*<0,0001).

To get a better insight into the nature of the immune response of mice infected with these isolates, spleen expression of IFN-γ, TNF and IL-4 was determined by real-time RT-PCR at one, three, and seven days post-infection (Fig. 5). Cytokine expression showed that at day three, mice infected with the 124A isolate presented significantly lower levels of IFN-γ in comparison with mice infected with 140A. However, by day seven post-infection this difference inverted, and mice infected with 124A presented significantly higher levels of IFN-γ and TNF (Fig. 5A and 5B). For expression of IL-4, no differences were found between the isolates, except at day seven post-infection, when isolate 140A resulted in the expression of slightly higher values (Fig. 5C).

### Resolution of inflammation is delayed in mice infected with the primary isolate

**Figure 6:**
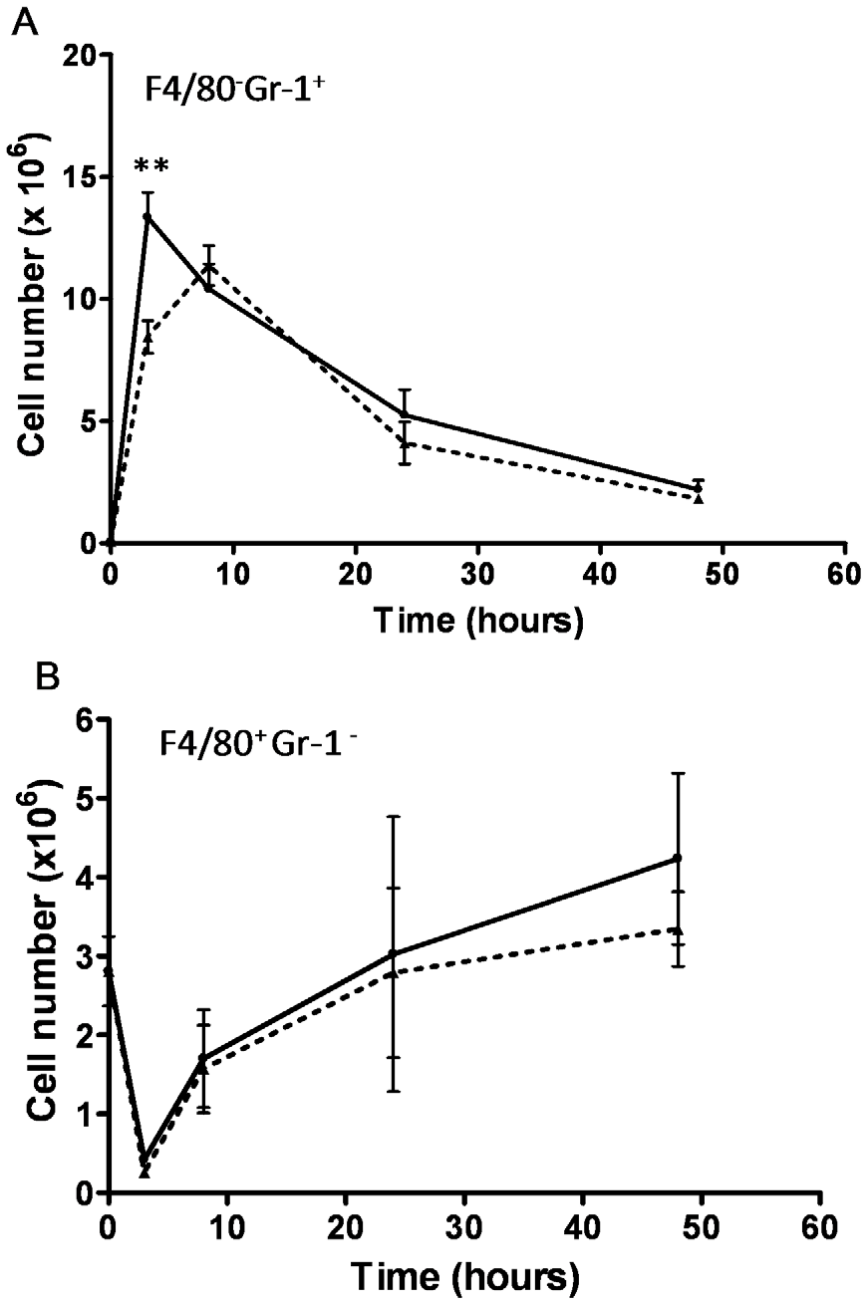
Intraperitoneal inflammatory response to *C. albicans* strain variants. Kinetics of neutrophils (A) and macrophages (B) in the peritoneal cavity following i.p. infection of BALB/c mice with 10^7^ cells from strains 124A (solid line) and 140A (dashed lines). Cells were recovered by peritoneal lavage, and counting of leukocytes was performed by flow citometry. Statistically significant differences between results at 3, 8, 24 and 48 hours of infection, as evaluated by Student's *t* test, are labeled with double asterisks (*P*<0.001).

A comparative analysis of leukocyte recruitment to the peritoneal cavity of mice infected with 124A or 140A was next performed. Figure 6 shows that *C. albicans* infection stimulated an acute leukocytosis, predominantly due to the recruitment of neutrophils, as previously described [33]–[35]. Counts of the peritoneal exudate leukocytes showed that the number of neutrophils increased more than 190 fold (*P*<0.001) in the infection with isolate 124A, and 158 fold (*P*<0.001) following infection with 140A. Using the set of resolution indices from Bannenberg *et al.* [36], in the time interval between three h (T_max_) and 20 h (T_50_), exudate PMNs decreased in number from 13.34×10^6^ (Ψ_max_) to 6.7×10^6^ (R_50_), resulting in a resolution interval (R_i_) of 17 h (i.e., 3–20 h), in the infection with isolate 124A. For mice infected with 140A, the Ψ_max_ was much lower, 11.38×10^6^, and T_max_ higher (eight hours), resulting in a resolution index of 13 h (i.e., 8–21 h). The macrophage cell population showed a similar kinetics in mice infected with isolates 124A and 140A (Fig. 6B). These results indicated that the two *C. albicans* isolates raise similar patterns of leukocyte recruitment. However, the resolution of inflammation is four hours delayed in mice infected with the primary isolate, 124A.

### Subsequent isolates induce reduced macrophage death

**Figure 7:**
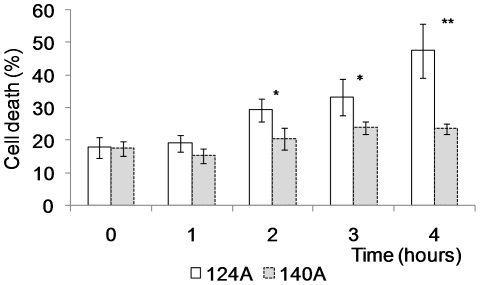
*In vitro C. albicans* macrophage killing. Cells from the macrophage cell line J774 were incubated with *C. albicans* isolates 124A or 140A cells in a ratio of 1∶5 (E∶T) and dead macrophages identified after incubation with 1 µg/ml of propidium iodide under the florescence microscope. Statistically significant differences between results at each hour of co-infection, as evaluated by Student's *t* test, are labeled with single asterisk (*P*<0.05).

Isolates 124A and 140A were tested *in vitro* with a macrophage cell line. The percentage of phagocytosis was approximately 11% for isolate 124A and 17% for 140A, but this difference was not statistically significant (*P* = 0.076). Phagocyte death, assessed by the number of PI-positive phagocytes, showed that isolate 124A induced death of about 50% of the macrophages after 4 h of co-incubation (Fig. 7). On the contrary, 140A did not induce a significant change in the percentage of macrophage death during the same period. Differences in macrophage death induced by both isolates were statistically significant after 2 h (*P* = 0.011) of co-incubation, and continued after 3 h (*P* = 0.037) and 4 h (*P* = 0.001).

### Subsequent isolates have different phenotypic characteristics regarding activity of secreted enzymes and response to stress agents

Phenotypic characteristics known to contribute to *C. albicans* pathogenicity, such as growth rate, response to stress and activity of extracellular enzymes, were evaluated in the two clinical isolates. No significant differences were observed regarding the ability of the isolates to secrete aspartic proteases (Saps) or in their growth rates at 26, 30 or 37°C in SD and YPD media (results not shown). The extracellular in vitro phospholipase activity, determined as the Pz value, showed that isolate 124A presented a higher activity than isolate 140A (Pz value of 0.52±0.001 for 124A and of 0.86±0.042 for 140A, *P*<0.05).

**Figure 8:**
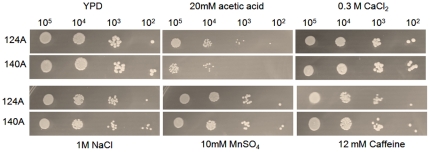
*In vitro* susceptibility assay. Growth of 124A and 140A yeast cells at 37°C for 48 h on YPD and YPD containing 20mM acetic acid, 0.3M CaCl_2_, 1M NaCl, 10mM MnSO_4_, and 12mM caffeine. Drop tests were performed by spotting 10 µl of 10^5^, to 10^2^ cells/ml dilutions.

The behaviour of both clinical isolates showed no significant differences regarding growth in the presence of CaCl_2_, Caffeine, MnSO_4_, SDS and ethanol, at all tested concentrations, as well as on SD plates at pH 3.7, pH 5.5 or pH 8.0. Both isolates seemed to be equally resistant to osmotic stress induced by NaCl (1M) and sorbitol (1.2 M). However, in the presence of 20 mM acetic acid, isolate 124A was more tolerant than isolate 140A (Fig. 8). The response to 1.25 mM H_2_O_2_ oxidative stress of the two isolates showed that 140A was significantly more sensitive to H_2_O_2_ induced death than isolate 124A, presenting a decrease in viability of around 50% [124A 98% (±23.3), 140A 52% (±2.3), *P*<0.05].

Phenotypic characterisation showed that the genetic variants behaved similarly although isolate 124A presented a higher phospholipase activity and was more tolerant to acetic acid and H_2_O_2_ than 140A.

## Discussion

Infections due to *C. albicans* may result from the selective proliferation of a single strain variant present in the commensal population before invasive infection [7], [8]. In patients with recurrent infections, three basic scenarios were described: (i) maintenance of that same strain, (ii) maintenance of that same strain undergoing microevolution or microvariation, or (iii) strain replacement [4], [6], [7], [8]. Microvariations are relatively frequent and may occur in response to changes within the host, reflecting strain adaptation. Therefore, with the characterization of strains sequentially isolated from patients with recurrent infections, it is important to evaluate whether these adaptations have consequences in host-pathogen interaction. These aspects are particularly relevant when dealing with commensal organisms. It has long been known that different *C. albicans* strains can exhibit varying levels of virulence when tested both *in vivo* and *in vitro* models [37]–[41]. However, to the best of our knowledge, no work has characterized the virulence of isolates, and their genetic variants, sequentially recovered from the same patient, as described in the present study.

In this work, four sequential isolates from a patient were genotyped by MLST and MLP and found to be closely related. Cluster analysis, including other strains isolated in the same period from other patients of the hospital, confirmed that the four isolates were very close since they were the only ones to group within a *p* distance value lower than 0.02. According to Odds *et al.* [7] strains that group within this *p* distance value could be considered undistinguishable or variants of the same strain. Interestingly, these isolates showed a clear progressive decrease in virulence in an i.v. mouse model of systemic infection. Since they were variants of the same strain, we concluded that the differences in virulence were not due to different genetic backgrounds of the isolates but to strain adaptation to host changes during the recurrent infections. One might doubt that these changes occurred in such a short time, however recent studies showed that *C. albicans* isolates undergo chromosomal and genetic alterations during a single passage in the mouse [9]. Following these results, the original isolate, 124A, and the first recurrent isolate to present a significant decrease in virulence (140A) were selected in order to understand the mechanisms underlying the observed differences in virulence.

Systemic infection by *C. albicans* is associated with the release of proinflamatory cytokines, including TNF and IFN-γ [42], [43]. In this study cytokine quantification showed that although at day seven post-infection the levels of IFN-γ and TNF increased in mice infected with 124A, on day three the levels of IFN-γ were lower, comparing with mice infected with 140A. Moreover, cells from isolate 124A developed long filaments inside the kidney, while cells from 140A appeared as fragmented hyphae intermixed with the inflammatory cells. These observations are in agreement with previous reports, indicating that pro-inflammatory cytokines are important for antifungal effector functions, particularly during the early phase of the inflammatory response [12], [33], [34], [44], [45].

The differences observed in the murine virulence study could be due to a differential recognition of the isolates by the host cells, resulting in an impaired inflammatory cellular response, or to an intrinsic higher resistance of isolate 124A to phagocyte killing.

The comparative analysis of leukocyte recruitment to the peritoneal mouse cavity of mice infected with 124A or 140A showed that even though resolution of inflammation was delayed in mice infected with 124A, immunocompetent mice recognized both isolates similarly, invoking an acute neutrophilia, as previously described [35], [46], [47]. Thus, we tested the hypothesis that the differences in virulence observed in infected mice could be mainly due to an intrinsically higher resistance to phagocyte killing of isolate 124A. One mechanism proposed for the opportunistic *C. albicans* to resist phagocyte killing is by rapidly changing to a filamentous form, allowing the fungal cells to resist ingestion or, if internalized, kill the phagocyte to escape to the extracellular environment [29], [30]. This higher resistance was confirmed *in vitro* upon co-incubation with J774 macrophages cell line. Both isolates were equally recognized and phagocytosed, but 124A cells induced a much higher macrophage death than 140A cells. The observation that 124A resisted more efficiently to the presence of H_2_O_2_, a compound present in the hostile environment of the phagolysosome, and presented a higher activity of secreted phospholipases, also favored its resistance to phagocyte induced death.

Overall, this comparative analysis demonstrated that the virulence decrease of isolate 140A was related to its lower ability to resist to anticandida effector mechanisms, what explains the lower kidney CFU's, the absence of long filaments in kidney histology and in vitro assays, and the faster spontaneous resolution of acute inflammation. We believe that the four isolates from this patient are genetic variants of a strain that, upon interaction with the host, adapted to differences in the microenvironment. It is likely that, as the patient became immunocompromised, the host environment became less stressful, and adaptation resulted in a progressive decrease in virulence that was evidenced when tested in an immunocompetent host. Several works analyzing rates of genetic and genomic alterations and their possible consequences to microbe fitness propose that for opportunistic pathogens, such as *C. albicans*, these alterations favor the commensal state rather than the infectious [9], [48], [49]. Additionally, Cheng *et al.* [50] isolated a *C. albicans* variant with attenuated virulence after passages through mice, which could also be considered in agreement with the commensal theory proposed.

This study is the first to show a decrease in virulence of genetic variants of the same strain sequentially isolated from a human patient, suggesting that *C. albicans* is able to adjust to the host, favoring commensalism rather than increase of virulence. The ability of *C. albicans* to adapt to and change its virulence in immunocompromised hosts can be a strategy of this organism to maintain its host alive and prolong its own survival.

## Materials and Methods

### Yeast strains and typing

*C. albicans* clinical strains (14 isolates) used in this study were collected from nine patients with recurrent infections attending the same hospital (Table 1). The four isolates analysed in more detail were from a patient with gastro-intestinal cancer who had been under chemotherapy (patient 1). This patient was submitted to surgical intervention and presented two sequential bloodstream infections in a period of four months (Table 1). All the isolates and the reference strain SC5314, were maintained on Sabouraud agar plates at 4°C and cryopreserved in 30% glycerol (wt/wt) at −80°C.

Strain typing was performed by using microsatellite length polymorphism (MLP) and multilocus sequence typing (MLST), the more discriminatory typing methods for C. albicans. For MLP analysis polymerase chain reaction (PCR) amplification with CAI, CAVI, and CEF3 markers was performed as described by Sampaio *et al.* [6] and by Bretagne *et al.* [51]. PCR products were run in an ABI 310 Genetic Analyser (AB Applied Biosystems) and fragment sizes were determined automatically using the GeneScan 3.7 analysis software. MLST typing was based on sequence analysis of DNA fragments from the six housekeeping *C. albicans* genes, *ACC1*, *ADP1*, *GLN4*, *RPN2*, *SYA1*, and *VPS13*, as previously reported [52]. The diploid sequence types (DST) obtained were deposited in the *C. albicans* MLST database (http://calbicans.mlst.net/).

Similarities between MLST sequence data were analyzed in terms of *p* distance with MEGA version 4.0 [53], as described by Odds *et al.* [7]. Nodal support, after 1000 bootstrap replications, was also calculated and depicted in the UPGMA dendrogram.

#### Mice and *C. albicans* hematogenously disseminated infection

Female BALB/c mice 6 to 8 weeks old were obtained from Charles River (Barcelona, Spain) and kept under specific pathogen-free conditions at the Animal Facility of Life and Health Sciences Research Institute (Braga, Portugal). The present study was conducted under the guidelines and approval of the Research Ethics Committee of the same Institute.

To evaluate the virulence of the isolates mice were injected intravenously (i.v.) in the lateral tail vein with 2×10^6^ cells of each of the four isolates studied in more detail, in 0.5 ml PBS. For preparation of inocula, cells unfrozen from the original stock were grown in Winge medium (0.2% glucose and 0.3% of yeast extract) at 26°C, to maintain the conidial morphology [54]. In each experiment, all isolates were tested simultaneously and inocula were confirmed by CFU counting of the suspensions used to infect mice. Animal welfare was assessed twice daily during 30 days.

For assessment of organ fungal-burdens and cytokine quantification mice were separated in groups, four mice in each cage, and i.v. infected with a lower inoculum, 10^6^ yeast cells. At days 1, 3, and 7 post-infection, mice from a cage were sacrificed and their kidneys, livers, and spleens aseptically processed. Organs were homogenized in 2 ml of Hanks Balanced Salt Solution (HBSS) from Invitrogen, diluted, and cultured on Sabouraud agar at 37°C. The results of organ fungal burden were expressed as log CFU/ml of homogenate. Prior to processing, spleens were divided in half to allow simultaneous analysis of fungal colony counts and cytokine quantification.

### Cytokine quantification

RNA was isolated from the spleen homogenate obtained previously. Briefly, 200 µl of the organ homogenate were centrifuged at 6000 rpm at 4°C and the pellet resuspended in 0.5 ml of Trizol reagent. After 5 minutes of incubation at room temperature 0.1 ml of chloroform was added, tubes were agitated and incubated on ice for 15 minutes. Samples were then centrifuged at 12000 g for 15 minutes at 4°C and the aqueous phase recovered. RNA was precipitated from the aqueous phase by mixing with isopropyl alcohol, and samples centrifuged at 12000 g for 10 minutes at 4°C. RNA pellet was washed once with 0.8 ml of 70% ethanol and air-dried. RNA was resuspended in 10 µl of ultra-pure water, quantified in the NanoDrop 1000 R Spectrophotometer (NanoDrop Technologies, Inc., Wilmington, NC), and stored at −80°C at a concentration of 200 ng/µl.

Total RNA was reverse transcribed in a thermocycler My Cycler Thermal Cycler (Bio-Rad, Hercules, CA) by using the Superscript Kit II and Oligo dT (Invitrogen). The cDNA was subjected to real-time RT-PCR reactions for quantification of mRNA levels of TNF, IFN-γ, IL-4, and the housekeeping gene mHPRT by using the LightCycler (Roche, Basel, Switzerland), and the LightCycler FastStart DNA Master Hybridization Probes kit. Probes and primer sequences used to amplify the cDNA, as well as the specific annealing temperatures are described in Botelho *et al.* [55].

### Histology

Kidneys excised from infected mice were fixed in 10% phosphate-buffered formalin, embedded in paraffin, sectioned, and stained with periodic acid-Schiff (PAS) stain after hematoxylin-Eosin (H&E) staining, according to Kretschmar *et al.* [56].

### Quantification of *in vivo* acute inflammatory response

To quantify the cellular acute inflammatory response to isolates 124A or 140A, mice were intraperitoneally (i.p.) injected with 10^7^ *C. albicans* cells and killed after 3, 8, 24 and 48 h [56]. The inflammatory infiltrate was collected by lavage with ice-cold PBS [57]. Quantification of leukocyte sub-populations in the peritoneal lavage fluids was performed by flow cytometric analysis (FACScan) based on the expression of F4/80, a marker associated with the macrophage lineage [58], and GR1, a marker associated primarily with the granulocyte lineage [59]. The following monoclonal antibodies (mAbs) were used in the cytometric analysis (Becton-Dickinson, San Jose, CA) using CELLQUEST software (Becton-Dickinson): Phycoerythrin (PE) conjugated anti-mouse F4/80 antigen (clone BM8), and FITC anti-mouse Ly-6G and Ly-6C (Gr-1) (RB6-8C5) (BD Pharmingen). To characterize the resolution of inflammation the following quantitative indices were used: (i) the magnitude of PMN tissue infiltration (maximal PMN, Ψ_max_); (ii) the time interval when numbers of PMN reach Ψ_max_ within exudates (T_max_); (iii) the time point (T_50_) when PMN numbers reduce to 50% of Ψ_max_ (R_50_); and (iv) the resolution interval (R_i_), the time interval from the maximum PMN point (Ψ_max_) to the 50% reduction point (R_50_) [i.e.T_50_-T_max_] [36].

### Macrophage culture and phagocytosis assays

The mouse macrophage-like cell line J774 (ATCC TIB-67) was cultured at 37°C in 5% CO_2_ in Dulbecco's Modified Eagle's medium (DMEM), supplemented with 10% heat-inactivated fetal calf serum (FCS) (Valbiotech), 2 mM L-glutamine, 1 mM sodium pyruvate, and 10 mM HEPES. Macrophages were plated at a concentration of 5×10^5^ cells/ml into 24-well tissue culture plates (Orange) containing a 13 mm diameter coverslip (Nunc) in each well and incubated overnight in 5% CO_2_ at 37°C. *C. albicans* isolates were grown overnight at 26°C in Winge medium, recovered by centrifugation at 5000 rpm and washed twice in sterile phosphate buffered saline (PBS).

Phagocytosis was assessed at a 5∶1 *C. albicans*/macrophage ratio, and the number of internalized *C. albicans* cells determined in a phase-contrast microscope (Leica DMRB) after 30 minutes of co-incubation [60], [61]. Percentage of phagocytosis was determined as the number of internalized cells/number macrophages ×100. At least 300 cells were counted.

Macrophage death assessment was determined by incubating macrophages and yeast cells, as previously described, and cells stained with 1 µg/ml propidium iodide (PI) after 1, 2, 3, and 4 h of incubation. Images were taken in ten independent fields using a Leica DM5000B fluorescence microscope. Percentage of dead phagocytes was determined as the number of PI positive macrophages/number macrophages counted ×100 [62]. At least 300 cells were counted.

### Phenotypic screening and susceptibility assays

For the determination of growth rates, a pre-culture was prepared incubating *C. albicans* isolates over night at 30°C in liquid SD (0.17% of YNB, 0.5% of (NH_4_)_2_SO_4_, 2% of glucose) and YPD (1% yeast extract, 2% peptone, 2% glucose) media and then a dilution was prepared in fresh medium to start with a OD_600_ of 0.05. Growth rates were determined at 26°C, 30°C and 37°C by measuring OD_600_ every hour until the culture reached stationary phase.

Extracellular proteolytic activity (Saps) of the isolates was assessed in BSA solid and liquid medium according to Monod *el al.* [63]. *C. albicans* isolates were also screened for production of extracellular phospholipase activity by growing them on egg yolk agar and measuring the size of the zone of precipitation by the method of Samaranayake *et al.* [64]. Phospholipase activity (Pz value) was calculated as the ratio of the diameter of the colony and the diameter of the colony plus that of the precipitation zone. Since Saps and phospholipases are inducible enzymes these tests were performed with freshly unfrozen cells from the original stocks.

Strain sensitivity to osmotic, acidic and oxidative stress was determined by incubating the yeast cells on SD agar plates containing several stress conditions: 50 and 100 µg/ml SDS; 1 M NaCl, 7% and 11% Ethanol; 1.2 M Sorbitol; 20 and 50 mM Acetic Acid; 12 mM Caffeine; 0.3 M CaCl_2_; 10 mM MnSO_4_, 0.2 M LiCl; and also SD at pHs 3.75, 5.5 and 8. Cells grown overnight in YPD medium were recovered by centrifugation at 5000 rpm and washed in PBS. Drop tests were performed by spotting 10 µl of 10^5^ to 10^2^ cells/ml dilutions onto the prepared plates that were incubated at 26, 30 and 37°C, for 3 days. Susceptibility to H_2_O_2_ was assessed by incubating the yeast cells in a solution of 1.25 mM H_2_O_2_, for 60 minutes at 30°C, and viability measured by CFU counts on YPD D.

### Statistical analysis

Unless otherwise stated, results shown are from one experiment, representative of three independent experiments. Statistical significance of results was determined by unpaired Student t-test and survival data were analyzed with the log-rank test, using the GraphPad Prism 4 Software (GraphPad Software, Inc., La Jolla, CA, USA). Results were considered statistically significant with *P* values of less than 0.05.
